# Psychometric properties of JPSS (Japanese parenting style scale) between mother and father in West Java, Indonesia

**DOI:** 10.3389/fpsyg.2026.1785102

**Published:** 2026-05-13

**Authors:** Ratna Jatnika, Hendriati Agustiani, Yanti Rubiyanti, Syauqiyyah Syahlaa, Rahmad Muliadi, Fanisya Triputri Sahrani, Unique Nur Alifa Putri, Keisuke Okubo

**Affiliations:** 1 Department of Psychology, Faculty of Psychology, Universitas Padjadjaran, Bandung, Indonesia; 2 Center for Psychometrics Study, Faculty of Psychology, Universitas Padjadjaran, Bandung, Indonesia; 3 Department of Education, Faculty of Letters, Kokushikan University, Tokyo, Japan

**Keywords:** multiple indicators multiple causes, parenting styles, parents, reliability, validity

## Abstract

**Introduction:**

Accurate measurement of parenting styles is essential for understanding disciplinary practices and comparing mother and father contributions in Indonesian cultural context. In this context, Japanese Parenting Style Scale (JPSS) assesses warmth, hostility, permissiveness, and harsh control, but psychometric evidence for Indonesian adaptation and measurement equivalence between mothers and fathers remains limited. Therefore, this research aimed to evaluate the initial psychometric properties of Indonesian version of JPSS and examine potential parental differences and measurement bias.

**Methods:**

Participants were parents of preschool-aged children recruited from several cities in West Java, Indonesia. Confirmatory factor analysis (CFA) was used to test the factor structure, while omega coefficients were adopted to assess internal consistency. Multiple Indicators Multiple Causes (MIMIC) method was applied to detect differential item functioning (DIF) between mothers and fathers.

**Results:**

The four-factor structure was supported with necessary item refinements during the adaptation process. The indicators showed meaningful loadings on the respective constructs. Reliability estimates for all dimensions ranged from adequate to excellent. Comparative analyses indicated that mothers reported higher levels of warmth and hostility than fathers, while permissiveness and harsh control were relatively comparable. DIF analyses identified several items in the warmth dimension that functioned differently across parental status, showing potential measurement bias in raw score comparisons.

**Discussion:**

Indonesian version of JPSS showed promising psychometric properties for assessing parenting styles. However, mother–father comparisons were interpreted with caution due to item-level measurement nonequivalence. Future research could include more diverse samples, formally test measurement invariance, and incorporate qualitative methods to strengthen validity evidence.

## 1 Introduction

Discipline in parenting is crucial for several reasons, impacting children’s development and family dynamics. Effective discipline helps in improving children’s behavior by setting clear expectations and consistent consequences for misbehavior (Grusec et al., 2017; Koocher et al., 2024; Verser, 2014). This corrects undesirable behavior and promotes self-regulation and emotional control (Sangsuk and Thipchart, 2023). Discipline allows parents to teach societal values and norms, preparing children to function as responsible adults (Wissow, 2014). This includes guiding children to understand acceptable behaviors and the consequences of actions. Positive discipline strategies, such as reasoning and autonomy support, develop better emotional regulation and self-control (Grusec et al., 2017; Sangsuk and Thipchart, 2023). This is essential for overall mental health and the ability to handle stress (Wissow, 2014). Knowledgeable and consistent discipline practices contribute to better cognitive development in children. Parents who are well-informed about children’s development tend to use more effective and less harsh disciplinary methods (Mayer and Blome, 2013; Vally and El Hichami, 2020). Effective discipline contributes to a warm and stable family atmosphere, which is conducive to healthy growth (Verser, 2014). The concept promotes a sense of security and trust in the family unit. Positive discipline is built on a foundation of a warm and trusting relationship between parents and children. This includes clear communication, attention, and praise for good behavior, which strengthens parent–child bond (Koocher et al., 2024).

The roles of fathers and mothers in child-rearing are complementary and are essential for optimal children’s development. Mothers are often positioned as primary caregivers who provide sensitive and responsive care. Meanwhile, fathers tend to contribute through challenging forms of parenting and emotional support. Effective cooperation between fathers and mothers, as well as a holistic family-based method, is crucial for creating a caregiving environment that supports children’s developmental needs (Bentenuto and Venuti, 2019; Jeong et al., 2018; Lee et al., 2023; Pekel-Uludağlı, 2024; Rajhans et al., 2019; Steenhoff et al., 2021). Historically, fathers have been seen as the primary disciplinarians in the family, often associated with harsher and more aggressive disciplinary methods (Macleod and Lesch, 2024). This traditional view is evolving, with fathers increasingly adopting non-violent and gentle disciplinary methods (Macleod and Lesch, 2024). Mothers are often more included in day-to-day discipline, using positive and negative strategies. Existing research shows that mothers discipline children more frequently than fathers (Hallers-Haalboom et al., 2016; Wang et al., 2023). Positive discipline, which includes setting limits and providing structure, is more commonly used by mothers, specifically in early childhood (Wang et al., 2023). Fathers’ positive and negative discipline at an early age significantly predicts children’s behavior control (Wang et al., 2023). Harsh discipline can have long-term negative effects on children, including increased risk of violent attitudes and behaviors in adulthood (Sangsuk and Thipchart, 2023). Fathers’ harsh discipline is particularly connected to higher levels of children’s aggression (Lee et al., 2011).

The inclusion of fathers in parenting is crucial for positive children’s development. Factors such as mother gatekeeping and children’s temperament can influence the level of father inclusion. Effective discipline often requires cooperation between both parents. Consistent and supportive parenting contributes to better children’s adjustment and fewer behavioral problems (Cheung et al., 2018). In this context, fathers and mothers play critical roles in disciplining children, with the methods and participation influenced by traditional roles, societal changes, parental well-being, and cultural contexts. Effective discipline includes a balance of positive and negative strategies, cooperation between parents, and consideration of children’s age and gender. Understanding these dynamics can help in promoting healthier disciplinary practices and better children’s development outcomes.

Several measurement instruments have been developed to assess disciplinary practices, each differing in methodological approach and conceptual focus. Dimensions of Discipline Inventory (DDI) is a multidimensional instrument designed to evaluate a wide range of parental disciplinary methods, including corrective behaviors and compliance-oriented strategies. DDI has been validated across diverse populations, including university students in Spain, and supports a nine-factor structure organized into four second-order scales for assessing disciplinary practices (Gámez-Guadix et al., 2010). Another instrument, Harsh Discipline Preference Discrete Choice Experiment (HDP-DCE), uses image-based assessments to evaluate preferences for disciplinary strategies, with the aim of reducing response biases commonly associated with self-report surveys. This measure has shown excellent reliability and validity in the United States populations (Quick et al., 2023).

Parenting Scale (PS) is a widely used instrument for assessing dysfunctional parenting practices through subscales such as Laxness and Over-reactivity. Even though some ambiguity in the factor structure has been reported, PS remains a reliable tool for research purposes (Salari et al., 2012). Japanese Parenting Style Scale (JPSS) was developed to assess parenting styles in Japanese cultural context, focusing on dimensions such as warmth, hostility, permissiveness, and harsh control (Okubo et al., 2022). This scale facilitates a deeper understanding of culturally specific parenting practices in Japan and the implications for children’s development. The instruments provide a robust framework for evaluating diverse disciplinary practices and parenting styles across different cultural contexts.

JPSS, developed by Okubo et al. (2022), represents a valuable instrument for assessing parenting styles and the implications for disciplinary practices and children’s behavior. Japan is often characterized as a society that places strong emphasis on discipline, a value reflected across various aspects of daily life, including child-rearing practices. Parenting in Japan tends to emphasize a balance between warmth and control, with relatively prominent authoritarian practices. This method is characterized by high expectations and strict discipline to promote respect for authority and adherence to social norms (Hosokawa and Katsura, 2019; Okubo et al., 2022). Authoritarian parenting styles, including high levels of control and rule enforcement, have been shown to enhance children’s academic achievement motivation (Watabe and Hibbard, 2014). At a global level, Japan consistently ranks highly in educational achievement due to disciplined methods of parenting and education (Nakamura et al., 2020).

In the Indonesian context, empirical studies have shown that parenting practices related to warmth, discipline, and parental control reflect distinct cultural patterns. Indonesian parents often integrate emotional closeness with behavioral guidance, emphasizing respect, relational harmony, and situational flexibility in discipline (Febiyanti and Yulindrasari, 2021). Comparative cross-cultural work further indicates that Indonesia differs in several important ways from East Asian contexts, including Japan, particularly in terms of the balance between emotional expressiveness, parental authority, and expectations for children’s compliance. Japanese child-rearing emphasizes *amae* (emotional dependence) and *shitsuke* (internalized discipline), whereas Indonesian families combine communal responsibility with a growing openness to autonomy-supportive interactions (Cheung and Cheah, 2025; Rizki and Akbar, 2024). These cultural distinctions underscore the need to examine whether a scale developed in Japan, such as the JPSS, functions similarly when administered to Indonesian parents.

Since parenting styles are shaped by sociocultural values and norms, adapting the scale to Indonesian context is essential to ensure the relevance and applicability (Sumargi et al., 2014, 2015). For the scale to be effectively used across diverse Indonesian settings, the adaptation process must reflect culturally specific parenting practices as perceived by mothers and fathers. Differences between groups cannot be interpreted meaningfully if the measurement tool functions differently across parental roles. Invariance testing is particularly important in the Indonesian cultural context, where gendered expectations of parenting may influence how specific behaviors are interpreted and reported. Therefore, adapting the JPSS and evaluating its psychometric properties—including potential item-level biases—is an important step to ensure valid and comparable assessments of parenting practices among Indonesian mothers and fathers.

## 2 Methods

### 2.1 Participants

A total of 829 parents from Tasikmalaya, Bandung, and Cimahi, Indonesia, who had children aged 3–5 years, participated in this research. The sample was obtained using a convenience sampling method (Hair et al., 2019), and the sample size was considered sufficient for confirmatory factor analysis (CFA) (Muthén and Muthén, 2002). Participants completed the questionnaire and demographic information in an offline setting and provided informed consent. Information regarding the research objectives and participation procedures was communicated before data collection. A total of 547 (66%) and 282 (34%) participants were mothers and fathers, respectively. The ages ranged from 21 to 50 years, with a mean of 34 years (SD = 5.35). Ethical clearance was granted by the Research Ethics Committee of Universitas Padjadjaran (Approval No. 554/UN6. KEP/EC/2024).

### 2.2 Instrument

JPSS (Okubo et al., 2022) is a parenting style measurement instrument developed using theoretically grounded items designed to be easily understood by Japanese parents. This instrument consists of 28 items, including four factors in line with Baumrind (1967) classification of parenting styles. The first factor, labeled warmth (9 items), comprises items reflecting positive parenting practices and corresponds to the authoritative style identified in previous research (Robinson et al., 1995). The second factor, hostility (6 items), reflects the aggressive or emotional aspects of authoritarian parenting. Furthermore, the third factor, permissiveness (6 items), captures indulgent parenting behaviors. The fourth factor, harsh control (7 items), represents the strict and controlling dimension characteristic of authoritarian parenting (Okubo et al., 2022). The original JPSS was translated into Indonesian using a forward–backward translation procedure and evaluated for content validity by experts in psychology and psychological measurement. The adaptation process followed the guidelines established by International Test Commission (ITC) (2017). Each item was rated on a six-point Likert scale ranging from not true (1) to true (6).

### 2.3 Data analysis and procedures

CFA was conducted using Weighted Least Squares Mean and Variance adjusted (WLSMV) estimator (Muthén and Satorra, 1995; Muthén and Asparouhov, 2002) in Mplus version 8.6 (Muthén and Muthén, 2021) to evaluate the fit of the measurement model. Furthermore, model fit was assessed using multiple goodness-of-fit indices, including Comparative Fit Index (CFI) and Tucker–Lewis Index (TLI) with values ≥ 0.90, as well as Root Mean Square Error of Approximation (RMSEA) and Standardized Root Mean Square Residual (SRMR) with values ≤ 0.08 (Hu and Bentler, 1999; MacCallum et al., 1996). The sample was randomly divided into two independent subsamples for cross-validation of the model solution, which demonstrated improved model fit after respecification (Brown, 2015; Kline, 2023). Then, internal consistency was estimated using McDonald’s omega coefficient (*ω*) (McDonald, 1999) to address the limitations of Cronbach’s alpha, which assumed tau-equivalence.

The subsequent step comprised detecting differential item functioning (DIF) using Multiple Indicators Multiple Causes (MIMIC) method (Jöreskog and Goldberger, 1975). Although MIMIC modeling can only test two sources of invariance, namely indicator intercepts (to examine differential item functioning or item bias) and factor means (to assess population heterogeneity), MIMIC is a more parsimonious model because it uses a single input matrix and estimates fewer parameters independently. Measurement model parameters do not need to be estimated separately for each group, as in multi-group CFA (MGCFA) (Brown, 2015; Leitgöb et al., 2023).

CFA model served as the baseline before adding a covariate to represent parental status (dummy coded as father = 1, mother = 2). Direct effects of the covariate on latent factors were interpreted as evidence of population heterogeneity, reflecting differences in latent factor means across parental status. In contrast, direct effects of the covariate on observed indicators showed the differences in means across parental status (Brown, 2015). Modification indices and expected parameter changes were analyzed to identify significant direct effects, which were then added to the model. The magnitude of the direct effects was interpreted as 0.20 = small, 0.50 = moderate, and 0.80 = large (Brown, 2015; Cohen, 1992).

## 3 Results

Table 1 shows the stages of the CFA conducted in this study. The initial CFA on the first sample indicated that the model did not fit the data well. Model diagnostics based on the modification indices (MI) were examined, and four items, namely i18, i21, i23, and i26, were deleted due to potential cross-loadings on unintended factors. After item deletion, the revised 24-item model demonstrated acceptable model fit. This structure was then retested and was found to fit the data from the second sample. Table 2 presents a summary of the factor loadings and inter-factor correlations of the items for the Indonesian version of the JPSS. All remaining items loaded significantly on their respective latent constructs (*p* < 0.05) (Brown, 2015). Reliability analyses indicated omega coefficients (*ω*) of 0.92, 0.89, 0.70, and 0.79 for warmth, hostility, permissive parenting, and harsh control, respectively for full sample. All ω values fell in the adequate to excellent range (≥0.70; McDonald, 1999).

**Table 1:** Fit indices of CFA and MIMIC Model.

Model	*χ* ^2^	*df*	*p*	RMSEA [90% CI]	CFI	TLI	SRMR
CFA							
4 factors 28 items (*n* = 391)	1,545.98	344	<0.001	0.09 [0.09, 0.10]	0.81	0.80	0.11
4 factors 24 items (*n* = 391)	768.70	246	<0.001	0.07 [0.07, 0.08]	0.91	0.90	0.08
4 factors 24 items (*n* = 438)	781.16	246	<0.001	0.07 [0.06, 0.08]	0.92	0.92	0.07
MIMIC							
Zero direct effects (*n* = 829)	1,368.72	266	<0.001	0.07 [0.07, 0.07]	0.91	0.90	0.07
Free direct effects (*n* = 829)	1,337.06	263	<0.001	0.07 [0.07, 0.07]	0.91	0.90	0.07

**Table 2:** Standardized factor loadings and factor correlation matrix of the Indonesian JPSS.

Items	First run (*n* = 391)				Second run (*n* = 438)			
	Warmth	Hostility	Permissive parenting	Harsh control	Warmth	Hostility	Permissive parenting	Harsh control
i1	0.60^***^				0.66^***^			
i2	0.75^***^				0.78^***^			
i3	0.70^***^				0.82^***^			
i4	0.80^***^				0.76^***^			
i5	0.64^***^				0.71^***^			
i6	0.74^***^				0.73^***^			
i7	0.76^***^				0.65^***^			
i8	0.74^***^				0.79^***^			
i9	0.77^***^				0.74^***^			
i10		0.72^***^				0.70^***^		
i11		0.66^***^				0.75^***^		
i12		0.76^***^				0.81^***^		
i13		0.72^***^				0.79^***^		
i14		0.78^***^				0.82^***^		
i15		0.80^***^				0.76^***^		
i16			0.58^***^				0.61^***^	
i17			0.71^***^				0.73^***^	
i19			0.57^***^				0.63^***^	
i20			0.44^***^				0.51^***^	
i22				0.33^***^				0.37^***^
i24				0.51^***^				0.56^***^
i25				0.65^***^				0.68^***^
i27				0.86^***^				0.81^***^
i28				0.79^***^				0.75^***^
Warmth	1				1			
Hostility	−0.28^**^	1			−0.34^***^	1		
Permissive parenting	−0.06	0.64^***^	1		−0.11	0.52^***^	1	
Harsh control	0.65^***^	−0.02	0.15^**^	1	0.60^***^	−0.01	0.14^**^	1

^*^p < 0.05; ^**^p < 0.01; ^***^p < 0.001.

**Figure 1:**
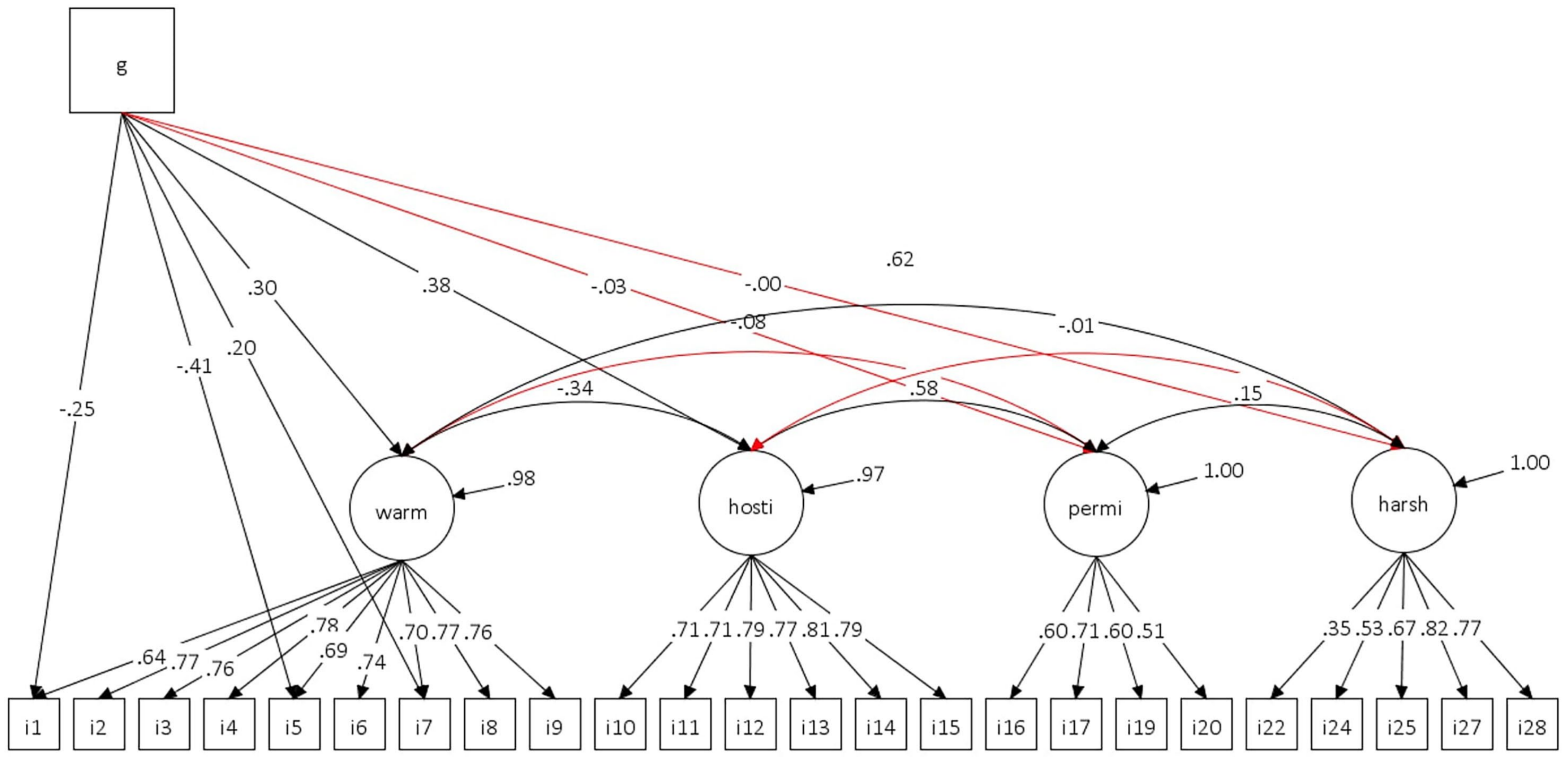
JPSS MIMIC model.

The MIMIC model with data from all respondents, in which all direct effects of the covariate on the indicators were constrained to zero, showed an acceptable fit to the data. Inspection of the modification indices suggested freeing the direct effects of the covariate on Items 1, 5, and 7. The fit indices remained satisfactory following model modification (Table 1). Figure 1 presents the path diagram of JPSS MIMIC model.

The regression paths connecting the covariate G (parental gender) to the latent variables warmth and hostility were statistically significant. Meanwhile, the paths to permissiveness and harsh control were not significant. The results showed that mothers exhibited higher mean levels than fathers on the latent factors. MIMIC model further reported that significant direct effects of G were observed on Items 1 and 5 in a negative direction, and on Item 7 in a positive direction, after controlling for the latent warmth factor. At the same level of the latent construct, fathers obtained lower scores on Items 1 and 5 by 0.25 and 0.41 units, respectively. On Item 7, mothers reported higher scores by 0.20 units compared to fathers.

## 4 Discussion

The initial JPSS model comprising 28 items did not meet acceptable criteria in Indonesian sample. After removing four items (i18, i21, i23, and i26) that showed indications of cross-loadings on unintended factors, the revised 24-item model reported adequate fit indices, with all remaining items meeting established psychometric criteria. The four-factor structure of JPSS is consistent with widely cited parenting style frameworks in the literature. However, the need to eliminate several items during the adaptation process shows the presence of cultural and semantic nuances influencing the reflection of latent constructs in Indonesian context.

The modification of the scale in this study, particularly the removal of several items during the split-sample CFA, was guided by both statistical fit considerations and theoretically informed cultural reasoning. We acknowledge that sample-specific factor structures are not merely statistical noise, but rather meaningful indicators of cultural (in)compatibility (Henrich et al., 2010). In the present study, the discarded items demonstrated a lack of conceptual equivalence within the Indonesian context, suggesting that the original measurement model may be partially misspecified for non-WEIRD populations. Although the core theoretical framework remained robust through cross-validation, we emphasize that in cases of more substantial cultural divergence, reconducting an Exploratory Factor Analysis (EFA) is necessary to ensure ecological validity and to avoid prioritizing theoretical purity over cultural relevance.

A closer examination of the four deleted items suggests that their poor psychometric performance was likely driven by cultural and semantic factors that reduced their conceptual distinctiveness in the Indonesian context. Item 18 and Item 21 were originally intended to capture permissiveness. However, Indonesian parents commonly interpret “membantu anak” or “memaafkan tanpa marah” as expressions of warmth, support, or parental responsibility rather than lenient control. As a result, both items tended to overlap with the warmth dimension, leading to cross-loadings. Item 23 was designed to reflect harsh control, but within Indonesian cultural norms, telling a child to stop crying can be perceived as a practical, situational instruction rather than a stable disciplinary style. This dual interpretation caused the item to correlate with both harsh control and firm control. Finally, Item 26 also showed conceptual ambiguity; while intended to represent harsh control, the phrase “marah besar” may reflect momentary emotional reactivity rather than a consistent disciplinary strategy. In collectivistic Indonesian families, such emotional displays can be viewed as spontaneous expressions of frustration rather than deliberate control, resulting in cross-loadings with permissiveness and inconsistent associations with harsh control. Together, these cultural and linguistic nuances likely contributed to multidimensional interpretations of the four items, leading to cross-loadings and supporting their removal to preserve factorial clarity in the Indonesian version of the JPSS.

The relatively lower reliability of the permissiveness factor may also be attributable to its small number of items. In classical test theory, reliability is strongly influenced by the length of a scale, with shorter subscales tending to show lower internal consistency even when the items are conceptually coherent (Crocker and Algina, 2008; Furr, 2022). Because the permissiveness factor contains only four items—considerably fewer than the warmth and control dimensions—the reliability estimate is inherently constrained. This structural limitation likely contributed to the lower level of internal consistency observed in our results. Future adaptations may benefit from expanding the number of items to capture a broader range of lenient parenting behaviors within the Indonesian context.

Inter-factor correlations provide insight into distinctive patterns of parenting practices. The negative association between warmth and hostility is consistent with the perspective that emotional warmth tends to inhibit aggressive or hostile expressions (Baumrind, 1966; Khaleque and Rohner, 2002; Rohner, 2004). In line with the results from Japanese validation research, warmth and hostility were negatively correlated (Okubo et al., 2022). The magnitude of the correlation was lower in Indonesian sample. In contrast, the moderate positive correlation between warmth and harsh control challenges the classical dichotomy between warmth and strictness. However, its magnitude remains within the acceptable threshold for inter-factor discriminant validity (< 0.85; Brown, 2015). This pattern reflects a culturally specific “authoritative” configuration in collectivistic contexts, in which emotional closeness coexists with firm boundary enforcement to maintain family harmony (Kagitcibasi, 2017). The positive association between hostility and permissiveness suggests the possibility of inconsistent parenting patterns, where parents alternate between hostile responses and leniency, creating ambiguity for children in understanding behavioral rules (Dwairy, 2010). Harsh control showed reduced association with hostility since strict rule enforcement is not necessarily accompanied by negative emotional expressions. Firmness is enacted in a relatively “cold” and consistent manner without hostility in with the distinction between behavioral and psychological control in parenting regulation literature (Barber, 1996; Steinberg et al., 1989). The small but significant correlation between permissiveness and harsh control suggests situational overlap. In this context, parents are permissive in certain domains while remaining strict regarding specific norm violations (Smetana, 2010).

The results showed that mothers exhibited higher mean latent scores than fathers on Warmth and Hostility factors. However, no significant differences were observed for the other two factors. These differences are attributable to socially ascribed parental roles, where fathers and mothers are more commonly positioned as primary breadwinners and caregivers, responsible for household and child-rearing tasks, respectively (Bemmelen et al., 2015; Istiyati et al., 2020). Law No. 1 of 1974 on Marriage stipulates in Article 34 paragraph (1) that “the husband is obliged to protect the wife and provide all necessities of household life according to the ability,” and in paragraph (2) that “the wife is obliged to manage household affairs to the best of the ability” (Tim, 1974). The normative and legal expectations contribute to parenting practices in Indonesia, placing greater demands on mothers’ inclusion in daily caregiving and disciplinary interactions (Istiyati et al., 2020).

Higher levels of warmth and hostility suggest that mothers tend to continue expressing emotional warmth in parenting, even when control is occasionally transferred through hostile responses. This pattern contrasts with fathers, who are more frequently perceived as figures of control, discipline, and firmness, and tend to display emotional warmth less often. The condition is consistent with the phenomenon of fatherlessness reported in Indonesia, where fathers are physically present but psychologically absent, leading children to experience control more prominently than emotional warmth (Ashari, 2018).

A different pattern has been reported in research conducted in Japan. In this context, Japanese fathers and mothers show distinctions between hostility and harsh control. Generally, women report higher levels of hostility, trait anger, or aggressive parenting tendencies than men (Okubo et al., 2022). Warmth exhibited the highest average score, followed by hostility and harsh control, which possessed relatively similar levels. Permissiveness showed the lowest mean score, but no specific data were reported regarding father–mother differences for each factor.

MIMIC model analysis identified DIF in warmth dimension based on parental gender for Items 1, 5, and 7. These results indicate that the instrument is not fully measurement invariant since fathers and mothers responded differently despite having equivalent levels of latent warmth (Brown, 2015). At the same level, fathers exhibited lower scores on Items 1 (“*Berusaha bermain dengan anak*”) and 7 (“*Menghabiskan waktu sebanyak mungkin dengan anak*”). This disparity reflects enduring social constructions of traditional gender roles, in which mothers are more often positioned as primary caregivers, while fathers tend to be more concentrated in provider roles (Lamb, 2010). Fathers’ lower scores on aspects related to time investment and intensity of play are influenced by historically reduced levels of paternal inclusion in terms of duration compared to mothers. According to Craig (2006), mothers continue to carry a greater share of childcare responsibilities characterized by direct interactive engagement, even though fathers’ inclusion increases over time. Fathers are more often included in supportive or play-related activities of shorter duration.

DIF on Item 5 (“*Tetap tenang dan peduli saat anak menangis*”) showed that fathers tended to obtain lower scores than mothers at equivalent levels of latent warmth, with a difference of 0.41 units. This result does not necessarily reflect lower paternal concern, but may capture differences in parenting self-efficacy. Mothers can develop adaptive emotion regulation and a deeper understanding of children’s non-verbal cues, such as crying, through socialization processes and more intensive caregiving experiences. In contrast, fathers may feel less competent in emotionally stressful child-related situations due to less frequent independent exposure (Coleman and Karraker, 2003; Parke and Cookston, 2019).

Although the MIMIC model identified DIF in three warmth items, these findings should be interpreted cautiously. The present data do not allow definitive conclusions about the underlying causes of DIF, and the observed patterns may reflect differences in response tendencies or item interpretation rather than actual differences in parenting behaviors. Accordingly, the explanations offered in this section are best viewed as theoretically informed interpretations rather than definitive empirical evidence. The absence of qualitative information regarding how fathers and mothers interpret specific warmth items limits the extent to which sociocultural explanations can be substantiated. Future research incorporating cognitive interviewing or mixed-methods approaches is needed to determine whether the DIF reflects true variations in parental warmth expression or measurement artifacts resulting from translation or contextual factors. This more cautious interpretation aligns with best practices in measurement invariance research, emphasizing that item-level non-equivalence must be acknowledged but should not be overinterpreted without convergent evidence.

The presence of DIF across the three items has important implications for the validity of total score comparisons. Mothers may systematically appear “warmer” simply because the items are more sensitive to maternal behavioral patterns, rather than reflecting essential differences in emotional capacity when raw total scores are used (Millsap, 2011). Therefore, these results show the importance of applying the latent variable modeling method or implementing corrections for biased item parameters in gender-comparative research. The conclusions regarding differences in paternal and maternal parenting practices are at risk of being distorted by measurement bias without the adjustment (Brown, 2015).

## 5 Practical implication

The findings of this study provide several implications for the use of the Indonesian JPSS in research and assessment practices. First, the presence of DIF in several warmth items indicates that direct comparisons of mothers’ and fathers’ raw scores may be biased, highlighting the need to use latent variable modeling or item-adjusted scores when analyzing gender differences. Second, the moderate association between warmth and harsh control suggests that Indonesian parents may integrate emotional closeness and firm structure in culturally specific ways, emphasizing the importance of interpreting these constructs within local sociocultural frameworks. Third, the lower reliability of the permissiveness factor, partly due to its smaller number of items, suggests that researchers should exercise caution when drawing conclusions from this dimension and consider supplementing the assessment of permissiveness in future work. Overall, the practical implications of this study primarily concern the psychometric application, interpretation, and refinement of the Indonesian JPSS rather than prescribing specific parenting practices.

## 6 Limitation

This research has several limitations despite the important contributions in Indonesian context. In addition to reliance on self-report data susceptible to social desirability bias, the discussion is largely inductive in the interpretation of statistical results, particularly concerning DIF and inter-factor correlations. This inductive method has limitations in terms of generalizability since the interpretations may not fully capture the diversity of parenting dynamics present in more heterogeneous Indonesian populations or in families with more progressive structures. The interpretations remain tentative due to unidentified sociocultural factors without direct confirmation through qualitative data from participants. Therefore, future research must adopt mixed methods to validate the psychological and semantic explanations underlying the quantitative results.

## 7 Conclusion

In conclusion, Indonesian version of JPSS shows promising psychometric properties for assessing parenting styles. However, mother–father comparisons should be interpreted with caution due to item-level measurement nonequivalence. Future research must include more diverse samples, formally test measurement invariance, and incorporate qualitative methods to strengthen validity evidence.

## Data Availability

The datasets presented in this article are not readily available because the original contributions presented in this research are included in the article/supplementary material, and further inquiries can be directed to the corresponding author. Requests to access the datasets should be directed to Ratna Jatnika, ratna@unpad.ac.id.

## References

[ref1] Ashari Y. (2018). Fatherless in Indonesia and its impact on children’s psychological development. Psikoislamika: Jurnal Psikologi Dan Psikologi Islam 15:35. doi: 10.18860/psi.v15i1.6661

[ref2] Barber B. K. (1996). Parental psychological control: revisiting a neglected construct. Child Dev. 67, 3296–3319. doi: 10.1111/j.1467-8624.1996.tb01915.x 9071782

[ref3] Baumrind D. (1966). Effects of authoritative parental control on child behavior. Child Dev. 37, 887–907. doi: 10.2307/1126611

[ref4] Baumrind D. (1967). Child care practices anteceding three patterns of preschool behavior. Genet. Psychol. Monogr. 75, 43–88, 6032134

[ref5] Bemmelen S. van T. Soesman M. Noya S. D. (2015). State of the World’s Fathers Country Report: Indonesia 2015. Jakarta: Rutgers WPF Indonesia.

[ref6] Bentenuto A. Venuti P. (2019). From supporting to co-parenting: the new roles of fathers. Parenting 19, 30–33. doi: 10.1080/15295192.2019.1555423

[ref7] Brown T. A. (2015). Confirmatory Factor Analysis for Applied Research. New York: Guilford Publications.

[ref8] Cheung R. Y. Boise C. Cummings E. M. Davies P. T. (2018). Mothers’ and fathers’ roles in child adjustment: parenting practices and mothers’ emotion socialization as predictors. J. Child Fam. Stud. 27, 4033–4043. doi: 10.1007/s10826-018-1214-1

[ref9] Cheung H. S. Cheah C. S. (2025). The forms and functions of parental control and parental warmth across cultures: evidence for commonality and specificity. Int. J. Behav. Dev. 49, 419–430. doi: 10.1177/01650254251337734

[ref10] Cohen J. (1992). A power primer. Psychol. Bull. 112, 155–159. doi: 10.1037/0033-2909.112.1.155, 19565683

[ref11] Coleman P. K. Karraker K. H. (2003). Maternal self-efficacy beliefs, competence in parenting, and toddlers’ behavior and developmental status. Infant Mental Health J. Off. Publ. World Assoc. Infant Mental Health 24, 126–148. doi: 10.1002/imhj.10048

[ref12] Craig L. (2006). Does father care mean fathers share? A comparison of how mothers and fathers in intact families spend time with children. Gender Soc. 20, 259–281. doi: 10.1177/0891243205285212

[ref13] Crocker L. M. Algina J. (2008). Introduction to Classical and Modern Test Theory. Noida: Cengage Learning.

[ref14] Dwairy M. (2010). Parental inconsistency: a third cross-cultural research on parenting and psychological adjustment of children. J. Child Fam. Stud. 19, 23–29. doi: 10.1007/s10826-009-9339-x

[ref15] Febiyanti A. Yulindrasari H. (2021). Cultural hybridity in parenting in Indonesia. 5th International Conference on Early Childhood Education (ICECE 2020), 163–167.

[ref16] Furr R. M. (2022). Psychometrics: An Introduction. 4th Edn Cambridge: SAGE publications.

[ref17] Gámez-Guadix M. Orue I. Calvete E. Carrobles J. A. Muñoz-Rivas M. Almendros C. (2010). Propiedades psicométricas de la versión española del Inventario de Dimensiones de Disciplina (DDI) en universitarios. Psicothema 22, 151–156. 20100442

[ref18] Grusec J. E. Danyliuk T. Kil H. O’Neill D. (2017). Perspectives on parent discipline and child outcomes. Int. J. Behav. Dev. 41, 465–471. doi: 10.1177/0165025416681538

[ref19] Hair J. F. Black W. C. Babin B. J. Anderson R. E. (2019). Multivariate data analysis. 8th Edn Noida: Cengage Learning.

[ref20] Hallers-Haalboom E. T. Groeneveld M. G. Van Berkel S. R. Endendijk J. J. Van Der Pol L. D. Bakermans-Kranenburg M. J. . (2016). Wait until your mother gets home! Mothers’ and fathers’ discipline strategies. Soc. Dev. 25, 82–98. doi: 10.1111/sode.12130

[ref21] Henrich J. Heine S. J. Norenzayan A. (2010). The weirdest people in the world? Behav. Brain Sci. 33, 61–83. doi: 10.1017/S0140525X0999152X, 20550733

[ref22] Hosokawa R. Katsura T. (2019). Role of parenting style in children’s Behavioral problems through the transition from preschool to elementary school according to gender in Japan. Int. J. Environ. Res. Public Health 16:21. doi: 10.3390/ijerph16010021, 30577659 PMC6339084

[ref23] Hu L. T. Bentler P. M. (1999). Cutoff criteria for fit indexes in covariance structure analysis: conventional criteria versus new alternatives. Struct. Equ. Model. Multidiscip. J. 6, 1–55. doi: 10.1080/10705519909540118

[ref24] International Test Commission (2017). ITC Guidelines for Translating and Adapting Tests. 2nd Edn Cambridge: Taylor & Francis.

[ref25] Istiyati S. Nuzuliana R. Shalihah M. (2020). Gambaran Peran Ayah dalam Pengasuhan. Profesi (Profesional Islam): Media Publikasi Penelitian 17, 12–19. doi: 10.26576/profesi.v17i2.22

[ref26] Jeong J. Siyal S. Fink G. McCoy D. C. Yousafzai A. K. (2018). “His mind will work better with both of us”: a qualitative study on fathers’ roles and coparenting of young children in rural Pakistan. BMC Public Health 18:1274. doi: 10.1186/s12889-018-6143-9, 30453979 PMC6245824

[ref27] Jöreskog K. G. Goldberger A. S. (1975). Estimation of a model with multiple indicators and multiple causes of a single latent variable. J. Am. Stat. Assoc. 70, 631–639. doi: 10.1080/01621459.1975.10482485

[ref28] Kagitcibasi C. (2017). Family, Self, and Human Development Across Cultures: Theory and Applications. 1st Edn Cambridge: Routledge.

[ref29] Khaleque A. Rohner R. P. (2002). Perceived parental acceptance-rejection and psychological adjustment: a meta-analysis of cross-cultural and intracultural studies. J. Marriage Fam. 64, 54–64. doi: 10.1111/j.1741-3737.2002.00054.x

[ref30] Kline R. B. (2023). Principles and Practice of Structural Equation Modeling. 5th Edn New York: The Guilford Press.

[ref31] Koocher G. P. La Greca A. M. Moorehead-Slaughter O. Lopez N. N. (2024). The Parents’ guide to Psychological first aid: Helping children and Adolescents cope with Predictable life Crises. Oxford: Oxford University Press.

[ref32] Lamb M. E. (2010). The role of the Father in child Development. 5th Edn Hoboken: John Wiley & Sons.

[ref33] Lee S. J. Kim J. Taylor C. A. Perron B. E. (2011). Profiles of disciplinary behaviors among biological fathers. Child Maltreat. 16, 51–62. doi: 10.1177/1077559510385841, 21062788

[ref34] Lee Y. Kim K. Zeng S. Douglass A. (2023). Mother-father relationships and child social-emotional adjustment: mediation through maternal and paternal parenting. Early Child. Res. Q. 63, 15–23. doi: 10.1016/j.ecresq.2022.11.001

[ref35] Leitgöb H. Seddig D. Asparouhov T. Behr D. Davidov E. De Roover K. . (2023). Measurement invariance in the social sciences: historical development, methodological challenges, state of the art, and future perspectives. Soc. Sci. Res. 110:102805. doi: 10.1016/j.ssresearch.2022.102805, 36796989

[ref36] MacCallum R. C. Browne M. W. Sugawara H. M. (1996). Power analysis and determination of sample size for covariance structure modeling. Psychol. Methods 1, 130–149. doi: 10.1037/1082-989x.1.2.130

[ref37] Macleod G. Lesch E. (2024). “These are his rules, our rules”: constructions of fathers as disciplinarians within low-income families of the Western cape. J. Comp. Fam. Stud. 54, 361–386. doi: 10.3138/jcfs.54.4.04

[ref38] Mayer L. M. Blome W. W. (2013). The importance of early, targeted intervention: the effect of family, maternal, and child characteristics on the use of physical discipline. J. Hum. Behav. Soc. Environ. 23, 144–158. doi: 10.1080/10911359.2013.747406

[ref39] McDonald R. P. (1999). Test Theory: A Unified Treatment. Mahwah: Erlbaum.

[ref40] Millsap R. E. (2011). Statistical Approaches to Measurement Invariance. New York: Taylor & Francis Group.

[ref41] Muthén B. Asparouhov T. (2002). Latent variable analysis with categorical outcomes: multiple-group and growth modeling in Mplus. Mplus web Notes 4, 1–22.

[ref42] Muthén L. K. Muthén B. O. (2002). How to use a Monte Carlo study to decide on sample size and determine power. Struct. Equ. Model. Multidiscip. J. 9, 599–620. doi: 10.1207/S15328007SEM0904_8

[ref43] Muthén L. K. Muthén B. O. (2021). Mplus (Version 8.6). [Statistical software]. Los Angeles: Muthén and Muthén.

[ref44] Muthén B. O. Satorra A. (1995). Technical aspects of Muthén’s LISCOMP approach to estimation of latent variable relations with a comprehensive measurement model. Psychometrika 60, 489–503. doi: 10.1007/BF02294325

[ref45] Nakamura R. Yamashita J. Akabayashi H. Tamura T. Zhou Y. (2020). A comparative analysis of children’s time use and educational achievement: assessing evidence from China, Japan and the United States. Chin. J. Sociol. 6, 257–285. doi: 10.1177/2057150X20911871

[ref46] Okubo K. Tang Y. Lee J. Endo T. Nozawa S. (2022). Development of the Japanese parenting style scale and examination of its validity and reliability. Sci. Rep. 12:18099. doi: 10.1038/s41598-022-23153-5, 36302842 PMC9613974

[ref47] Parke R. D. Cookston J. T. (2019). “Fathers and families,” in Handbook of Parenting: Volume 3: Being and Becoming a Parent, ed. Bornstein M. H.. 3rd ed (Cambridge: Routledge), 64–136.

[ref48] Pekel-Uludağlı N. (2024). The role of the father involvement in child development: the relationships with maternal, paternal, and child characteristics. Curr. Psychol. 43, 4085–4097. doi: 10.1007/s12144-023-04649-3

[ref49] Quick K. N. Vissoci J. R. Green E. P. Chase R. M. Puffer E. S. (2023). Adaptation and evaluation of a picture-based measure of parent discipline. J. Child Fam. Stud. 32, 2901–2914. doi: 10.1007/s10826-023-02640-x

[ref50] Rajhans P. Goin-Kochel R. P. Strathearn L. Kim S. (2019). It takes two! Exploring sex differences in parenting neurobiology and behaviour. J. Neuroendocrinol. 31:e12721. doi: 10.1111/jne.12721, 31034670 PMC6773483

[ref51] Rizki S. P. Akbar Z. (2024). Parent-child relationship in foreign and Indonesian families cross-cultural studies: a review. Proc. Ser. Psychol. 2, 331–333.

[ref52] Robinson C. C. Mandleco B. Olsen S. F. Hart C. H. (1995). Authoritative, authoritarian, and permissive parenting practices: development of a new measure. Psychol. Rep. 77, 819–830. doi: 10.2466/pr0.1995.77.3.819

[ref53] Rohner R. P. (2004). The parental “acceptance-rejection syndrome”: universal correlates of perceived rejection. Am. Psychol. 59, 830–840. doi: 10.1037/0003-066X.59.8.830, 15554863

[ref54] Salari R. Terreros C. Sarkadi A. (2012). Parenting scale: which version should we use. J. Psychopathol. Behav. Assess. 34, 268–281. doi: 10.1007/s10862-012-9281-x

[ref55] Sangsuk N. Thipchart T. (2023). Discipline strategies: parent’s experiences for early childhood development in north eastern, Thailand. Jurnal Keperawatan Soedirman 18, 8–17. doi: 10.20884/1.jks.2023.18.1.6618

[ref56] Smetana J. G. (2010). Adolescents, Families, and Social Development: How Teens Construct Their Worlds. Hoboken: John Wiley & Sons.

[ref57] Steenhoff T. Tharner A. Vaever M. S. (2021). Internalizing and externalizing problems in preschool children: the role of mothers’ and fathers’ observed parenting behavior in a well-resourced sample. Scand. J. Psychol. 62, 374–385. doi: 10.1111/sjop.12724, 33719054

[ref58] Steinberg L. Elmen J. D. Mounts N. S. (1989). Authoritative parenting, psychosocial maturity, and academic success among adolescents. Child Dev. 60, 1424–1436. doi: 10.2307/1130932, 2612251

[ref59] Sumargi A. Sofronoff K. Morawska A. (2014). Parenting practices and parenting programs in Indonesia: a literature review and current evidence. ANIMA Ind. Psychol. J. 29, 186–198.

[ref60] Sumargi A. Sofronoff K. Morawska A. (2015). Understanding parenting practices and parents’ views of parenting programs: a survey among Indonesian parents residing in Indonesia and Australia. J. Child Fam. Stud. 24, 141–160. doi: 10.1007/s10826-013-9821-3

[ref61] Tim B. I. P. (1974). Undang-Undang Republik Indonesia Nomor 1 Tahun 1974 tentang Perkawinan. Jakarta: Bhuana Ilmu Populer.

[ref62] Vally Z. El Hichami F. (2020). Knowledge about parenting as a predictor of behavioral discipline practices between mothers and fathers. Psychol. Stud. 65, 40–50. doi: 10.1007/s12646-019-00497-z

[ref63] Verser J. (2014). ““Time out”—calming the Chaos,” in 101 More Interventions in Family Therapy, eds. Nelson T. S. Trepper T. S. (Cambridge: Routledge), 233–241.

[ref64] Wang Z. Li W. Woudstra M. L. Wang L. (2023). Toddlers’ self-regulation development from 14 to 26 months: the unique role of paternal discipline. Infant Child Dev. 32:e2455. doi: 10.1002/icd.2455

[ref65] Watabe A. Hibbard D. R. (2014). The influence of authoritarian and authoritative parenting on children’s academic achievement motivation: a comparison between the United States and Japan. North Am. J. Psychol. 16, 329–338.

[ref66] Wissow L. (2014). “Corporal punishment and children’s mental health: opportunities for prevention,” in Violence and Mental Health: Its Manifold Faces, ed. Faces I. M. (Dordrecht: Springer Netherlands), 123–131.

